# A Rare Association of Compound Odontome with Missing Lateral Incisor

**DOI:** 10.5005/jp-journals-10005-1234

**Published:** 2014-04-26

**Authors:** Rangeeth Bollam Nammalwar, Joyson Moses

**Affiliations:** Reader, Department of Pedodontics and Preventive Dentistry Thai Moogambigai Dental College and Hospital, Chennai Tamil Nadu, India; Professor, Department of Pedodontics, Thai Moogambigai Dental College, Chennai, Tamil Nadu, India

**Keywords:** Odontoma, Compound odontoma, Odontogenic tumors, Impacted tooth, Missing tooth

## Abstract

Odontomas are a common type of odontogenic tumor, usually asymptomatic and mostly detected on routine radiographic examination. An 11-years-old male child with the chief complaint of mobility of deciduous dentition in the upper front region was diagnosed with an odontome with an impacted central incisor, missing lateral incisor and retained deciduous incisors following radiographic analysis. Histopathology revealed a compound odontoma following a conservative enucleation. Odontomas associated with primary dentition, impacted teeth and erupting into oral cavity have been described, but the association with a missing lateral incisor makes this an interesting case report.

**How to cite this article: **Nammalwar RB, Moses J. A Rare Association of Compound Odontome with Missing Lateral Incisor. Int J Clin Pediatr Dent 2014;7(1):50-53.

## INTRODUCTION

Odontomas are a common type of odontogenic tumors and are considered to be developmental anomalies (hamartomas) rather than true neoplasm. Fully developed odontomas consist mainly of enamel and dentin and variable amounts of cementum and pulp tissue.^1^ Complex odontomas are malformations in which all dental tissues are present, but arranged in a more or less disorderly pattern and compound odontomas represent malformations in which all of the dental tissues are represented in a pattern that is more orderly than that of the complex type similar to that of a natural tooth.^2^ Enamel, dentin, cementum and pulp are arranged as they would be in the normal tooth. The cells of the tissues in odontomas are normal but lack organization due to disordered expression and localization of the extra-cellular matrix molecules in the dental mesenchyme.^3^

The World Health Organization histological typing of odontogenic tumors classifes odontoma under benign tumors containing odontogenic epithelium with odonto-genic ectomesenchyme, with or without dental hard tissue formation. Ameloblastic fbro-odontoma, compound and complex odontoma are entities under this category.^4^ Hitchin suggested that odontomas are inherited through a mutant gene or interference, possibly postnatal, with genetic control of tooth development. In humans, there is a tendency for the lamina between the tooth germs to disintegrate into clumps of cells. The persistence of a portion of lamina may be an important factor in the etiology of complex or compound odontomas and either of these may occur instead of a tooth.^5^Experimental production of odontoma in rats as a result of trauma was studied by Levy BA suggestive of trauma as an etiological factor.^6,7^

They are mostly asymptomatic, but a study of 60 cases in Department of Oral Pathology, Dankook University Hospital between 1991 and 2006 revealed delayed eruption of either the deciduous or permanent tooth; intra- or extraoral swelling; and the reporting of pain. Eighteen cases had no subjective symptoms and of the 60 cases, 55% were female.^8^The anterior maxilla holds a somewhat stronger tendency for being the predilection site for compound odontoma than the posterior mandible does as the predilection site for the complex odontoma.^9^ The radiographic features of the odontoma show two regions, the well defined periphery which may be smooth or irregular, mostly with a hyper-ostotic or cortical border and a soft tissue capsule adjacent to the cortical border. The internal structure is largely opaque with compound odontoma showing a number of teeth like structures. The degree of opacity is equivalent or exceeds the adjacent structures.^10^

## CASE REPORT

**Fig. 1:**
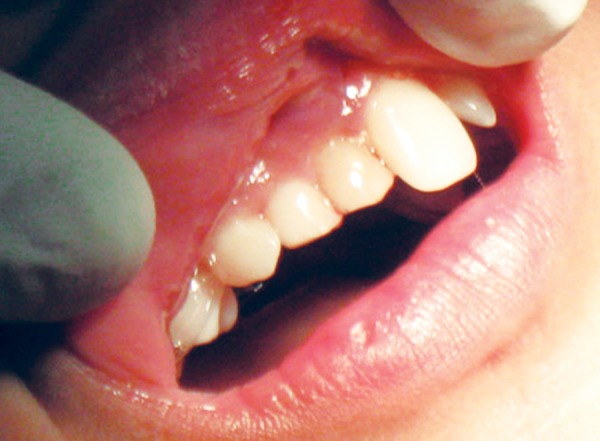
Preoperative view—retained deciduous incisors, missing permanent incisors

**Fig. 2:**
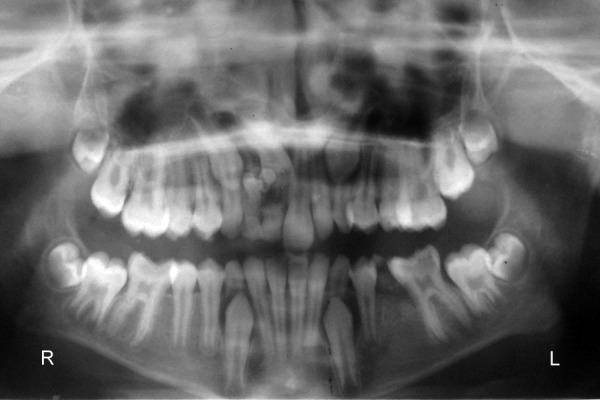
Panoramic radiograph showing a single calcified mass

**Fig. 3:**
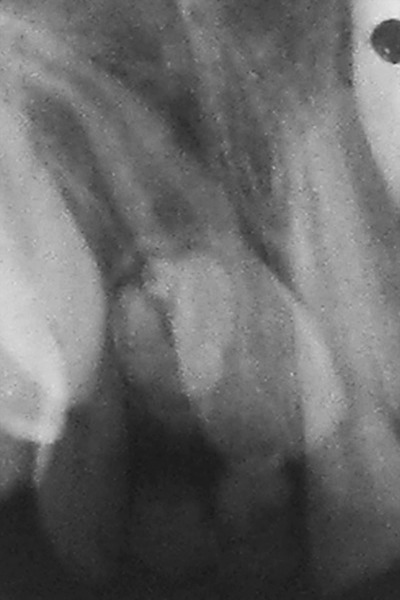
Periapical radiograph showing a single lesion of compound odontoma

**Fig. 4:**
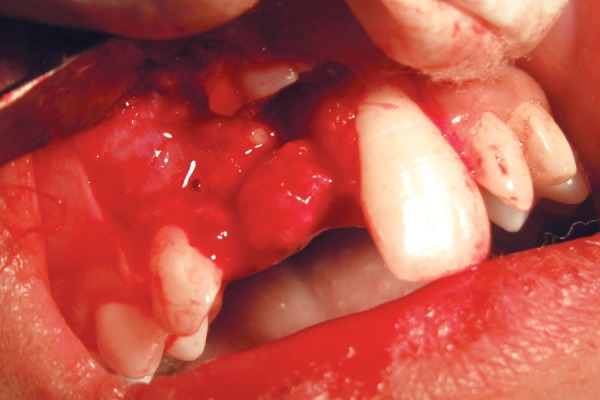
Mass of soft tissue seen superficial to the odontome

**Fig. 5:**
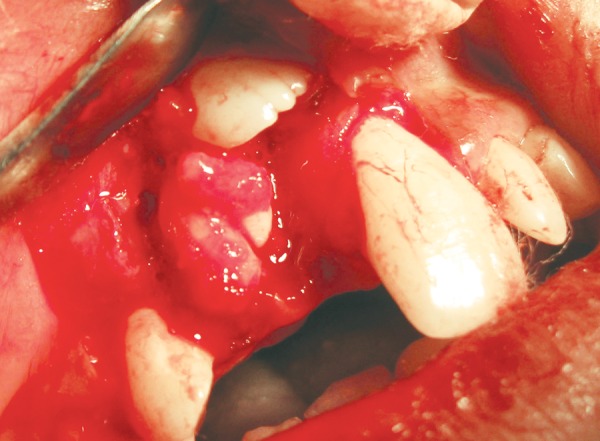
Exposure of the odontoma following removal of soft tissue

**Fig. 6:**
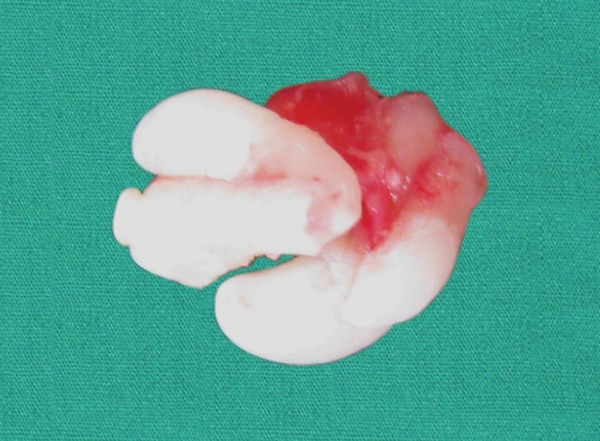
Odontoma after complete enucleation

An 11-years-old male child presented to the department of pedodontics with the chief complaint of mobile deciduous teeth in the right maxillary anterior region for the past 4 days with pain, with the history of dental trauma. Clinical examination of the maxillary anterior region revealed retained missing right maxillary permanent central and lateral incisor with retained deciduous incisors (Fig. 1). Panoramic radiograph (Fig. 2) revealed that a mass of radiopaque structure was present in proximity crown of the maxillary right central incisor which was impacted. The root formation of the impacted central incisor was complete at that time. The absence of the maxillary lateral incisor was clearly noted. Periapical radiograph of the maxillary right incisor region showed that multiple radiopaque structures were presented adjacent to the crown of the impacted central incisor and mesial to the canine (Fig. 3). A provisional diagnosis of an odontoma was made, and the patientwas scheduled for conservative surgical enucleation of the lesion. A mass of soft tissue 5 mm by 5 mm (Fig. 4) in the path of approach to the odontoma was excised and sent for histopathology along with the odontoma (Figs 5 and 6). The patient was treated under local anesthesia with no premedication and the behavior was definitely positive under the Frankel's behavior rating scale.

**Fig. 7:**
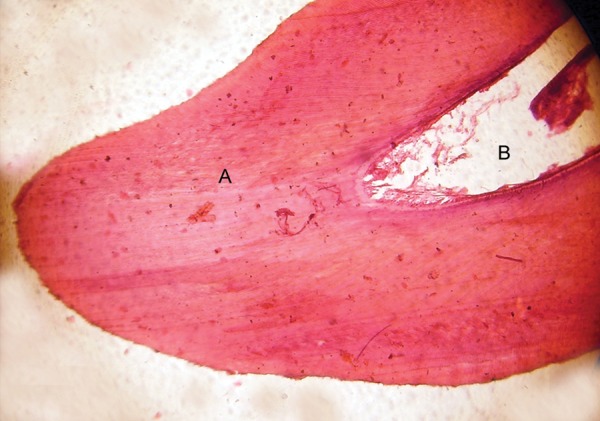
Decalcified section of the odontome showing (A) dentinal tubules, (B) pulpal space

**Fig. 8:**
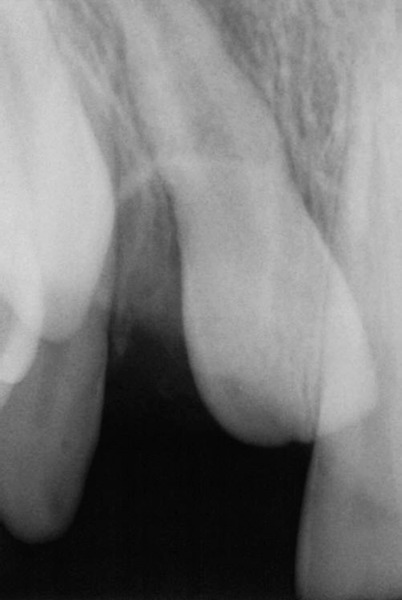
Postoperative radiograph after one week

Histopathology of the soft tissue section showed delicate cellular fbrous connective tissue, with dense focal collection of chronic infammatory cells like lymphocytes and plasma cells in few areas. Few islands of odontogenic epithelial cells were seen. The mass of hard tissue was confirmed to be a compound odontoma with the decalcified section (Fig. 7) showing dentinal tubules and pulp space as in the case of a normal tooth. The patient had an uneventful recovery and a periapical radiograph taken one week later shows no undue changes (Fig. 8). The patient was advised orthodontic extrusion of the impacted central incisor.

## DISCUSSION

Odontoma is a condition in dental medicine that mostly proceeds unrecognized until the occurrence of clinical symptoms such as delayed eruption, or is incidentally detected on routine X-ray examination. The exact cause is not known, however, previous dental trauma and infection have been postulated as the potential factors in the development of odontogenic tumor as described in the literature below.

The case described in this study was diagnosed initially as odontoma as the radiographic examination showed calci-fication similar to that of teeth. Histological examination of the lesion after enucleation revealed a compound odontoma and the surrounding mass of tissue could possibly be from the connective tissue surrounding the odontoma. The etio­logy of trauma being a causative factor as suggested by Levy BA^6,7^ could possibly be associated with this case. Trauma in the form of dental mutilation as a result of traditional tooth extractions a practice of the people of Africa have reported to cause malformation and dilacerations of permanent teeth.^11^Intrusion of the permanent dentition as a result of trauma leading to malformation in the form of hyperplasia of the permanent lateral incisor have been reported. The odontoma was reported to be located very deep for surgical removal to be carried out.^12^ The effects of odontoma on the primary dentition range from noneruption to impactions as in this case of an unerupted primary canine that was managed surgically with the unerupted tooth retained to allow its eruption.^13,14^

Our case history had a positive note on a traumatic epi­sode and considering the study of the literature available, further research into how, for example any histological or chemical as a result of trauma should be considered. The presence of the missing permanent lateral incisor is of course a unique entity and it may be theoretically considered that trauma can be an etiologic factor. Spontaneous eruption of an impacted tooth after removal of a supernumerary tooth or odontoma depends on several factors, such as distance of the apex of the impacted tooth relative to its midline, time of surgery relative to the expected eruption time of the impacted teeth and loss of space, estimated position, depth of impaction, angle of impaction relative to the midline, time of surgery relative to the expected eruption time of the impacted teeth.^15^ The treatment of choice for these impacted teeth associated to odontomas appears to be removal of the lesion with preservation of the impacted tooth. The latter in turn require clinical and radiological follow-up for at least one year. If no changes in the position of the tooth are seen during this period, fenestration followed by orthodontic traction is indicated. Extraction advised when the tooth is ectopic or heterotopic, with morphological alterations, or presence of cystic lesions.^16^ Orthodontic therapy for alignment of the impacted central incisor has been suggested.

## CONCLUSION

The detection of odontoma is more likely an accidental radio­logical finding, hence the need for routine radiographic analysis should be emphasized. Early diagnosis of odon-tomas in primary dentition is essential in order to prevent later complications, such as impaction or failure of eruption of teeth.
